# Supplementary material for: Skin Barrier Function and Microtopography in Patients with Atopic Dermatitis
Source: J Clin Med. 2024 Oct 1;13(19):5861. doi: 10.3390/jcm13195861 (PMC11477937; doi:10.3390/jcm13195861)
Supplement: Supplementary file 1 [file jcm-13-05861-s001.zip › jcm-3190518-supplementary.pdf]

Table S1. Analysis of Sociodemographic Characteristics, Severity, and Barrier Function and Topographic Parameters between Men and Women.

Variables	Men (n=12)	Women (n=20)	P-value
Age	36.83 (14.75)	27.35 (13.11)	0.07
Education Level			0.09
- Basic	16.7% (2)	35% (7)	
- Intermediate	58.3% (7)	20% (4)	
- Higher	25% (3)	45% (9)	
Place of Residence			0.27
- Urban	83.3% (10)	95% (19)	
- Rural	16.7% (2)	5% (1)	
Phototype			0.4
- I	16.7% (2)	10% (2)	
- II	58.3% (7)	35% (7)	
- III	16.7% (2)	45% (9)	
- IV	8.3% (1)	10% (2)	
Smoking Habit			0.82
- Yes	16.7% (2)	20% (4)	
- No	83.3% (10)	80% (16)	
Number of Cigarettes/Day	1.67 (4.44)	1.05 (2.56)	0.62
Alcohol Consumption			0.11
- Yes	58.3% (7)	30% (6)	
- No	41.7% (5)	70% (14)	
Number of Alcohol Units/Week	2.17 (2.66)	0.9 (1.52)	0.15
Family History of Skin Diseases			0.92
- Yes	33.3% (4)	35% (7)	
- No	66.7% (8)	65% (13)	
Other Diseases			0.61
- Yes	33.3% (4)	25% (5)	
- No	66.7% (8)	75% (15)	
Daily Treatment for Other Diseases			0.61
- Yes	33.3% (4)	25% (5)	
- No	66.7% (8)	75% (15)	
Sun Exposure			0.47
- Yes	41.7% (5)	55% (11)	
- No	58.3% (7)	45% (9)	
Time of Sun Exposure (minutes)	80 (118.17)	49.5 (59.43)	0.42
Use of Sunscreen			0.03
- Yes	25% (3)	65% (13)	
- No	75% (9)	35% (7)	
Use of Moisturisers			0.02

- Yes	75% (9)	100% (20)	
- No	25% (3)	0% (0)	
Number of Times Moisturiser Used per Week	4.67 (3.11)	6.05 (1.64)	0.18
Age of Atopic Dermatitis Onset	15.75 (23.22)	9.15 (17.16)	0.4
Family Members with Atopic Dermatitis			0.36
- Yes	33.3% (4)	50% (10)	
- No	66.7% (8)	50% (10)	
Comorbidities (asthma, rhinitis, conjunctivitis)			0.31
- Yes	58.3% (7)	40% (8)	
- No	41.7% (5)	60% (12)	
Previous Treatment for Atopic Dermatitis			0.82
- Yes	83.3% (10)	80% (16)	
- No	16.7% (2)	20% (4)	
Current Treatment for Atopic Dermatitis			0.43
- Topical	33.3% (4)	40% (8)	
- Systemic	16.7% (2)	25% (5)	
- Biological	33.3% (4)	10% (2)	
- Anti-JAK	16.7% (2)	25% (5)	
Location of Measured Lesion			0.06
- Forearm	58.3% (7)	70% (14)	
- Trunk	25% (3)	0% (0)	
- No lesion	16.67% (2)	30% (6)	
SCORAD	47.98 (26.17)	31 (26.79)	0.09
ROS	3.75 (2.56)	2.45 (2.52)	0.17
EASI	20.08 (15.69)	9.62 (12.47)	0.05
IGA	2.67 (1.23)	1.65 (1.39)	0.05
POEM	14.42 (9.3)	10.7 (8.91)	0.27
DLQI	8.92 (6.46)	6.65 (6.98)	0.37
NRS Pruritus	5.91 (3.56)	4.55 (3.63)	0.32
NRS Sleep	5.27 (4)	2.5 (3.36)	0.05
Healthy Skin Temperature (ºC)	31.9 (0.93)	31.76 (1.36)	0.72
Eczema Temperature (ºC)	32.33 (0.84)	32.41 (1.3)	0.87

Healthy Skin Erythema (UA)	260.41 (83.08)	201.97 (61.37)	0.03
Eczema Erythema (UA)	415.98 (77.97)	335.65 (63.66)	0.01
Healthy Skin TEWL (g/h/m²)	15.75 (8.21)	12.17 (2.54)	0.17
Eczema TEWL (g/h/m²)	23.23 (8.5)	30.1 (13.32)	0.17
Healthy Skin SCH (UA)	32.61 (12.17)	31.45 (11.48)	0.79
Eczema SCH (UA)	21.52 (11.29)	19.44 (10.07)	0.64
Healthy Skin pH	5.34 (0.73)	5.49 (0.51)	0.52
Eczema pH	5.36 (0.69)	5.29 (0.47)	0.79
Healthy Skin Elasticity R0 (%)	0.35 (0.09)	0.34 (0.08)	0.65
Eczema Elasticity R0 (%)	0.32 (0.15)	0.35 (0.08)	0.57
Healthy Skin Elasticity R2 (%)	0.77 (0.09)	0.78 (0.15)	0.95
Eczema Elasticity R2 (%)	0.77 (0.12)	0.8 (0.08)	0.55
Healthy Skin Elasticity R7 (%)	0.64 (0.12)	0.66 (0.12)	0.54
Eczema Elasticity R7 (%)	0.52 (0.12)	0.59 (0.12)	0.19
Healthy Skin antioxidant capacity (UA)	5.77 (2.62)	5.16 (2.7)	0.54
Eczema antioxidant capacity (UA)	4.88 (1.28)	4.62 (1.84)	0.71
Healthy Skin Hardness (UA)	9.73 (4.92)	9.57 (4.66)	0.93
Eczema Hardness (UA)	19.13 (11.18)	13.34 (8.06)	0.18
Healthy Skin Friction (UA)	156.42 (85.77)	190.7 (128.4)	0.42
Eczema Friction (UA)	80.04 (24.67)	119.39 (59.75)	0.04
Healthy Skin Brightness (UA)	6.66 (0.79)	6.34 (0.64)	0.22
Eczema Brightness (UA)	5.81 (1.35)	6.03 (1.32)	0.69
Healthy Skin Indentometer (mm)	1.83 (0.43)	2.11 (0.35)	0.05

Eczema Indentometer (mm)	1.98 (0.58)	1.89 (0.34)	0.67
Healthy Skin Softness (SEsm)	225.16 (83.8)	218.43 (53.64)	0.78
Eczema Softness (SEsm)	346.67 (115.86)	295.78 (108.32)	0.28
Healthy Skin Roughness (Ser)	2.93 (2.14)	1.78 (0.6)	0.1
Eczema Roughness (Ser)	6.06 (8.66)	2.25 (2.54)	0.21
Healthy Skin Scaling (SEsc)	0.38 (0.63)	0.23 (0.21)	0.46
Eczema Scaling (SEsc)	7.68 (9.28)	4.06 (5.66)	0.29
Healthy Skin Wrinkles (SEw)	65.45 (16.81)	60.17 (13.76)	0.34
Eczema Wrinkles (SEw)	85.81 (31.59)	64.42 (18.16)	0.08
Healthy Skin Surface Area (%)	677.52 (58.18)	707.58 (96.33)	0.34
Eczema Surface Area (%)	816.51 (203.59)	850.17 (88.09)	0.63
Healthy Skin Volume (mm^2)	97.47 (15.71)	96.89 (14.26)	0.92
Eczema Volume (mm^2)	107.91 (33.22)	103.4 (25.61)	0.71
Healthy Skin Contrast (UA)	1.22 (0.29)	1.36 (0.39)	0.3
Eczema Contrast (UA)	1.95 (1.19)	2.07 (0.63)	0.78
Healthy Skin Entropy (UA)	1.49 (0.04)	1.48 (0.04)	0.55
Eczema Entropy (UA)	1.43 (0.06)	1.42 (0.03)	0.82
Healthy Skin Variance (UA)	4.79 (0.52)	5.05 (0.81)	0.32
Eczema Variance (UA)	6.05 (1.75)	6.34 (0.79)	0.64
Healthy Skin Homogeneity (UA)	11.84 (36.26)	1.34 (0.06)	0.34
Eczema Homogeneity (UA)	1.33 (0.09)	1.27 (0.05)	0.12
Healthy Skin Anisotropy (UA)	26.2 (9.58)	33.44 (14.92)	0.11
Eczema Anisotropy (UA)	25.43 (10.11)	40.69 (18.34)	0.02

Total Number of Cells (Healthy)	142.25 (32.28)	126.65 (26.5)	0.15
Total Number of Cells (Eczema)	107.1 (34.42)	104.36 (37.75)	0.86
Corneofix All (%) Healthy	48.69 (12.22)	51.34 (18.66)	0.63
Corneofix All (%) Eczema	38.44 (22.18)	34.58 (11.69)	0.63
Corneofix All (mm^2) Healthy	13.19 (3.13)	14.79 (5.37)	0.3
Corneofix All (mm^2) Eczema	11.07 (6.39)	9.96 (3.37)	0.63
Scaling Index Healthy	24.44 (7.5)	27.09 (10.29)	0.44
Scaling Index Eczema	21.65 (15.23)	17.73 (6.03)	0.46

p= p-value after chi-square test for qualitative and dichotomous variables and after Student's t-test for independent samples with the grouping variable sex.

Table S2. Analysis by average age

Variables	Age ≥31 (n=12)	Age <31 (n=20)	P
Sex			
- Male	58.3% (7)	25% (5)	0.06
- Female	41.7% (5)	75% (15)	
Level of education			
- Basic	25% (3)	30% (6)	0.8
- Medium	41.7% (5)	30% (6)	
- Higher	33.3% (4)	40% (8)	
Place of residence			
- Urban	83.3% (10)	95% (19)	0.27
- Rural	16.7% (2)	5% (1)	
Phototype			
- I	16.7% (2)	10% (2)	0.6
- II	41.7% (5)	45% (9)	
- III	25% (3)	40% (8)	
- IV	16.7% (2)	5% (1)	
Smoking habit			
- Yes	8.3% (1)	25% (5)	0.24
- No	91.7% (11)	75% (15)	
Number of cigarettes/day	0.42 (1.44)	1.8 (4.03)	0.18
Alcohol habit			
- Yes	41.7% (5)	40% (8)	0.93
- No	58.3% (7)	60% (12)	
Number of UBEs/week	2 (2.86)	1 (1.37)	0.28
History of skin disease			
- Yes	41.7% (5)	30% (6)	0.5
- No	58.3% (7)	70% (14)	
Other diseases			
- Yes	50% (6)	15% (3)	0.03
- No	50% (6)	85% (17)	
Daily treatment			
- Yes	58.3% (7)	10% (2)	0.01
- No	41.7% (5)	90% (18)	
Frequently exposed to sun			
- Yes	50% (6)	50% (10)	1
- No	50% (6)	50% (10)	
Sun exposure time (minutes)	75 (108.92)	52.5 (70.1)	0.48
Use of sunscreen			
- Yes	50% (6)	50% (10)	1
- No	50% (6)	50% (10)	
Use of moisturisers			

- Yes	75% (9)	100% (20)	0.02
- No	25% (3)	0% (0)	
Number of times moisturiser is used/week	4.67 (3.03)	6.05 (1.73)	0.17
Age of DA onset	25.75 (25.76)	3.15 (6.18)	0.01
Family members with DA			
- Yes	50% (6)	40% (8)	0.58
- No	50% (6)	60% (12)	
Comorbidities (asthma, rhinitis, conjunctivitis)			
- Yes	66.7% (8)	35% (7)	0.08
- No	33.3% (4)	65% (13)	
Previous treatment for DA			
- Yes	83.3% (10)	80% (16)	0.82
- No	16.7% (2)	20% (4)	
Current treatment for DA			0.05
- Topical	25% (3)	45% (9)	
- Systemic	25% (3)	20% (4)	
- Biological	41.7% (5)	5% (1)	
- Anti-JAK	8.3% (1)	30% (6)	
Where is the lesion to be measured			1
- Forearm	58.3% (7)	70% (14)	
- Trunk	8.3% (1)	10% (2)	
- No lesion	33.3% (4)	20% (4)	
SCORAD	39.65 (30.72)	36 (26.01)	0.72
ROS	2.83 (2.98)	3 (2.38)	0.86
EASI	16.1 (15.62)	12.01 (13.9)	0.45
IGA	2.17 (1.7)	1.95 (1.23)	0.7
POEM	13.58 (10.27)	11.2 (8.46)	0.48
DLQI	9 (9.02)	6.6 (5.05)	0.41
NRS Itch	5.64 (4.06)	4.7 (3.4)	0.52
NRS Sleep	5 (4.2)	2.65 (3.36)	0.13
Healthy temperature (ºC)	31.99 (1.21)	31.7 (1.21)	0.52
Eczema temperature (ºC)	32.06 (1.37)	32.51 (1)	0.38
Healthy erythema (UA)	252.53 (83.76)	206.7 (64.96)	0.09
Eczema erythema (UA)	422.54 (75.2)	347.12 (71.98)	0.03

Healthy TEWL (g/h/m²)	14.23 (6.98)	13.09 (4.67)	0.58
Eczema TEWL (g/h/m²)	19.66 (8.25)	30.36 (11.87)	0.04
Healthy SCH (UA)	39.59 (6.59)	27.26 (11.53)	<0.001
Eczema SCH (UA)	23.99 (9.49)	18.79 (10.66)	0.28
Healthy pH	5.34 (0.66)	5.49 (0.56)	0.51
Eczema pH	5.3 (0.44)	5.33 (0.61)	0.93
Healthy elasticity R0 (%)	0.36 (0.08)	0.33 (0.08)	0.45
Eczema elasticity R0 (%)	0.35 (0.16)	0.34 (0.09)	0.76
Healthy elasticity R2 (%)	0.77 (0.07)	0.78 (0.16)	0.9
Eczema elasticity R2 (%)	0.75 (0.1)	0.8 (0.1)	0.22
Healthy elasticity R7 (%)	0.64 (0.1)	0.66 (0.13)	0.65
Eczema elasticity R7 (%)	0.51 (0.1)	0.58 (0.13)	0.21
Healthy QT (UA)	5.26 (2.81)	5.46 (2.61)	0.84
Eczema QT (UA)	4.9 (1.31)	4.66 (1.74)	0.75
Healthy hardness (UA)	7.95 (3.15)	10.63 (5.21)	0.08
Eczema hardness (UA)	18.03 (11.53)	14.81 (9.07)	0.47
Healthy friction (UA)	227.9 (86.76)	147.81 (119.67)	0.05
Eczema friction (UA)	80.66 (19.56)	112.19 (57.92)	0.06
Healthy shine (UA)	6.53 (0.83)	6.42 (0.64)	0.67
Eczema shine (UA)	5.92 (1.77)	5.94 (1.13)	0.98
Healthy indentometer (mm)	2.16 (0.39)	1.92 (0.39)	0.1
Eczema indentometer (mm)	1.79 (0.65)	1.99 (0.35)	0.46
Healthy smoothness (SEsm)	230.97 (36.97)	214.94 (77.9)	0.44
Eczema smoothness (SEsm)	344.9 (101.23)	305.49 (117.07)	0.45
Healthy roughness (Ser)	2.06 (0.66)	2.3 (1.8)	0.59
Eczema roughness (Ser)	4.4 (7.89)	3.6 (5.4)	0.78

Healthy flaking (SEsc)	0.29 (0.26)	0.29 (0.5)	0.99
Eczema flaking (SEsc)	5.73 (6.3)	5.5 (8.02)	0.95
Healthy wrinkles (SEw)	64.15 (10.01)	60.95 (17.38)	0.51
Eczema wrinkles (SEw)	80.2 (27.47)	10.5 (26.16)	0.42
Healthy surface (%)	690.48 (65.12)	699.8 (95.43)	0.77
Eczema surface (%)	877.32 (212.43)	819.18 (109.83)	0.51
Healthy volume (mm^2)	100.5 (15.76)	95.07 (13.82)	0.32
Eczema volume (mm^2)	113.3 (25.73)	101.98 (29.54)	0.39
Healthy contrast (UA)	1.32 (0.27)	1.31 (0.41)	0.91
Eczema contrast (UA)	2.19 (1.33)	1.95 (0.67)	0.18
Healthy entropy (UA)	1.47 (0.02)	1.49 (0.05)	0.37
Eczema entropy (UA)	1.41 (0.06)	1.43 (0.04)	0.36
Healthy variance (UA)	4.94 (0.58)	4.97 (0.81)	0.92
Eczema variance (UA)	6.56 (1.85)	6.08 (0.95)	0.53
Healthy homogeneity (UA)	1.34 (0.05)	7.64 (28.09)	0.33
Eczema homogeneity (UA)	1.29 (0.08)	1.3 (0.08)	0.94
Healthy anisotropy (UA)	35.39 (16.96)	27.92 (10.39)	0.19
Eczema anisotropy (UA)	30.25 (15.81)	36.01 (17.65)	0.46
Total number of cells (healthy)	134.75 (31.8)	131.15 (28.47)	0.74
Total number of cells (eczema)	104.86 (25.95)	105.76 (39.68)	0.96
Corneofix all (%) (healthy)	57.25 (12.63)	46.21 (17.23)	0.06
Corneofix all (%) (eczema)	41.75 (21.36)	33.9 (14.25)	0.4
Corneofix all (mm^2) (healthy)	16.49 (3.64)	12.81 (4.75)	0.03
Corneofix all (mm^2) (eczema)	12.02 (6.16)	9.76 (4.1)	0.4

Scaling index (healthy)	30.26 (9.01)	23.6 (8.75)	0.05
Scaling index (eczema)	22.88 (14.65)	17.91 (8.81)	0.43

p= p-value after Student's t-test for independent samples dividing the population into two groups using as a cut-off point; the mean age which was 30.91, so the first group with n=12 had age ≥31 and the second with n=20 and age <31 years.

Table S3. Differences between patients with EASI≥21 and EASI<21.

Variables	EASI ≥21 (n=10)	EASI <21 (n=22)	P
Sex			
- Male	60% (6)	27.3% (6)	0.08
- Female	40% (4)	72.7% (16)	
Age	35.9 (14)	28.64 (14.15)	0.19
Education Level			
- Basic	10% (1)	36.4% (8)	0.1
- Medium	60% (6)	22.7% (5)	
- Higher	30% (3)	40.9% (9)	
Place of Residence			
- Urban	90% (9)	90.9% (20)	0.94
- Rural	10% (1)	9.1% (2)	
Phototype			
- I	10% (1)	13.6% (3)	0.97
- II	40% (4)	45% (10)	
- III	40% (4)	31.8% (7)	
- IV	10% (1)	9.1% (2)	
Smoking Habit			
- Yes	30% (3)	13.6% (3)	0.27
- No	70% (7)	86.4% (19)	
Cigarettes/day	2.3 (4.86)	0.82 (2.38)	0.38
Alcohol Habit			
- Yes	60% (6)	31.8% (7)	0.13
- No	40% (4)	68.2% (15)	
UBEs/week	2.1 (2.77)	1.05 (1.65)	0.19
Skin Disease History			
- Yes	10% (1)	45.5% (10)	0.05
- No	90% (9)	54.5% (12)	
Other Diseases			
- Yes	50% (5)	18.2% (4)	0.06
- No	50% (5)	81.8% (18)	
Daily Treatment			
- Yes	50% (5)	18.2% (4)	0.06
- No	50% (5)	81.8% (18)	
Frequently Sun Exposed			
- Yes	20% (2)	63.6% (14)	0.02
- No	80% (8)	36.4% (8)	
Sun Exposure Time (minutes)	24 (50.6)	77.73 (93.81)	0.05
Use of Sunscreen			
- Yes	30% (3)	59.1% (13)	0.13
- No	70% (7)	40.9% (9)	
Use of Moisturiser			
- Yes	80% (8)	95.5% (21)	0.16

- No	20% (2)	4.5% (1)	
Times Moisturiser Used/Week	4.5 (2.92)	6 (1.95)	0.16
Age of DA Onset	16.3 (23.09)	9.5 (17.91)	0.42
Family Members with DA			
- Yes	10% (1)	59.1% (13)	0.01
- No	90% (9)	40.9% (9)	
Comorbidities (asthma, rhinitis, conjunctivitis)			
- Yes	80% (8)	31.8% (7)	0.01
- No	20% (2)	68.2% (15)	
Previous DA Treatments			
- Yes	100% (10)	72.7% (16)	0.07
- No	0% (0)	27.3% (6)	
Current DA Treatment			
- Topical	20% (2)	45.5% (10)	0.11
- Systemic	30% (3)	18.2% (4)	
- Biological	40% (4)	9.1% (2)	
- Anti-JAK	10% (1)	27.3% (6)	
Where is the lesion to be measured?			
- Forearm	70% (7)	59.1% (13)	0.87
- Trunk	10% (1)	9.1% (2)	
- No lesion	10% (1)	31.8% (7)	
SCORAD	68.9 (8.09)	23.04 (19.86)	<0.001
ROS	5.9 (1.79)	1.59 (1.53)	<0.001
IGA	3.6 (0.7)	1.32 (0.99)	<0.001
POEM	21.9 (4.23)	7.64 (6.91)	<0.001
DLQI	13.6 (7.14)	4.73 (4.48)	<0.001
NRS Itch	8.22 (1.86)	3.73 (3.34)	<0.001
NRS Sleep	7 (3.12)	2.05 (3.05)	<0.001
Healthy Temperature (ºC)	31.86 (1.02)	31.79 (1.29)	0.88
Eczema Temperature (ºC)	32.17 (1.33)	32.5 (0.98)	0.49
Healthy Erythema (UA)	288.31 (84.68)	194.6 (47.91)	0.01
Eczema Erythema (UA)	376.69 (64.17)	364.58 (89.15)	0.7
Healthy TEWL (g/h/m2)	14.73 (5.97)	12.96 (5.43)	0.41
Eczema TEWL (g/h/m2)	23.26 (9.12)	29.63 (12.93)	0.21

Healthy SCH (UA)	29.37 (13.03)	33.02 (10.97)	0.42
Eczema SCH (UA)	19.36 (9.27)	20.87 (11.31)	0.74
Healthy pH	5.74 (0.74)	5.3 (0.48)	0.11
Eczema pH	5.81 (0.41)	5.02 (0.41)	<0.001
Healthy Elasticity R0 (%)	0.37 (0.07)	0.33 (0.08)	0.17
Eczema Elasticity R0 (%)	0.37 (0.13)	0.32 (0.1)	0.33
Healthy Elasticity R2 (%)	0.79 (0.07)	0.77 (0.15)	0.75
Eczema Elasticity R2 (%)	0.76 (0.11)	0.8 (0.09)	0.33
Healthy Elasticity R7 (%)	0.64 (0.11)	0.66 (0.12)	0.72
Eczema Elasticity R7 (%)	0.48 (0.13)	0.61 (0.1)	0.01
Healthy QT (UA)	6.27 (3.23)	4.98 (2.31)	0.28
Eczema QT (UA)	4.32 (1.99)	4.97 (1.34)	0.35
Healthy Hardness (UA)	7.51 (3.09)	10.59 (5.01)	0.04
Eczema Hardness (UA)	19.44 (10.92)	13.53 (8.51)	0.19
Healthy Friction (UA)	158.82 (80.14)	186.49 (127.23)	0.53
Eczema Friction (UA)	86.62 (31.93)	112.81 (59.1)	0.17
Healthy Shine (UA)	6.34 (1.01)	6.51 (0.54)	0.61
Eczema Shine (UA)	5.1 (0.67)	6.44 (1.35)	0.01
Healthy Indentometer (mm)	2.01 (0.39)	2 (0.42)	0.95
Eczema Indentometer (mm)	1.96 (0.54)	1.91 (0.41)	0.77
Healthy Smoothness (SEsm)	256.17 (90.25)	204.94 (44.14)	0.12
Eczema Smoothness (SEsm)	318.95 (83.09)	315.8 (129.06)	0.94
Healthy Roughness (Ser)	2.36 (2.25)	2.14 (1)	0.78
Eczema Roughness (Ser)	5.04 (7.31)	3.11 (5.31)	0.5
Healthy Scaling (SEsc)	0.49 (0.65)	0.2 (0.23)	0.2

Eczema Scaling (SEsc)	7.25 (10.31)	4.56 (5.19)	0.48
Healthy Wrinkles (SEw)	67.95 (18.55)	59.51 (12.6)	0.14
Eczema Wrinkles (SEw)	83.4 (34.86)	67.29 (18.44)	0.23
Healthy Surface (%)	719.83 (70.5)	685.61 (89.32)	0.3
Eczema Surface (%)	805.24 (142.54)	854.68 (147.41)	0.43
Healthy Volume (mm2)	97.28 (14.04)	97.03 (15.13)	0.97
Eczema Volume (mm2)	102.93 (35.92)	106.69 (24.16)	0.79
Healthy Contrast (UA)	1.41 (0.36)	1.26 (0.36)	0.29
Eczema Contrast (UA)	1.88 (0.81)	2.11 (0.94)	0.55
Healthy Entropy (UA)	1.46 (0.04)	1.49 (0.04)	0.07
Eczema Entropy (UA)	1.43 (0.04)	1.43 (0.05)	0.83
Healthy Variance (UA)	5.16 (0.62)	4.86 (0.75)	0.29
Eczema Variance (UA)	5.98 (1.26)	6.36 (1.28)	0.49
Healthy Homogeneity (UA)	13.91 (39.74)	1.35 (0.06)	0.34
Eczema Homogeneity (UA)	1.32 (0.09)	1.28 (0.07)	0.25
Healthy Anisotropy (UA)	25.71 (12.1)	33 (13.72)	0.16
Eczema Anisotropy (UA)	33.65 (13.58)	34.74 (19.21)	0.88
Total Number of Healthy Cells	128.2 (39.91)	134.46 (23.92)	0.65
Total Number of Eczema Cells	84 (28.48)	118.4 (33.9)	0.02
Corneofix all (%) Healthy	49.86 (14.01)	50.57 (17.65)	0.91
Corneofix all (%) Eczema	41.58 (19.56)	32.95 (14.17)	0.22
Corneofix all (mm2) Healthy	13.36 (3.7)	14.56 (5.08)	0.51
Corneofix all (mm2) Eczema	11.98 (5.64)	9.49 (4.08)	0.22
Scaling Index Healthy	25.75 (7.84)	26.25 (10.06)	0.89

Scaling Index Eczema	23.08 (13.55)	17.13 (8.38)	0.2
Corneofix all (%) Healthy	49.86 (14.01)	50.57 (17.65)	0.91
Corneofix all (%) Eczema	41.58 (19.56)	32.95 (14.17)	0.22
Corneofix all (mm2) Healthy	13.36 (3.7)	14.56 (5.08)	0.51
Corneofix all (mm2) Eczema	11.98 (5.64)	9.49 (4.08)	0.22

p= p-value after performing a Student's t-test for independent samples with the grouping variable EASI, constructing two groups using 21 as the cut-off point (indicating the point of progression from moderate to severe disease). The first group with n=10 had an EASI≥21 and the second group with n=22 had an EASI score<21.
